# Experiences and challenges of pre-exposure prophylaxis initiation and retention among high-risk populations: qualitative insights among service providers in Thailand

**DOI:** 10.3389/fpubh.2024.1366754

**Published:** 2024-05-15

**Authors:** Ajaree Rayanakorn, Sineenart Chautrakarn, Kannikar Intawong, Chonlisa Chariyalertsak, Porntip Khemngern, Debra Olson, Suwat Chariyalertsak

**Affiliations:** ^1^Department of Pharmacology, Faculty of Medicine, Chiang Mai University, Chiang Mai, Thailand; ^2^Faculty of Public Health, Chiang Mai University, Chiang Mai, Thailand; ^3^Division of AIDS and STIs, Department of Disease Control, Ministry of Public Health, Nonthaburi, Thailand; ^4^School of Public Health, University of Minnesota-Twin Cities, Minneapolis, MN, United States

**Keywords:** HIV, prevention, pre-exposure prophylaxis, PrEP, service provider, Thailand

## Abstract

**Objectives:**

Pre-exposure prophylaxis (PrEP) has been an essential element of the national combination prevention package and included in the Universal Health Coverage (UHC) of Thailand since 2019. As a part of the national monitoring and evaluation framework, this qualitative study aims to describe experiences and barriers concerning PrEP initiation and retention among service providers from both hospital and Key Population Led Health Service (KPLHS) settings under the country’s UHC roll-out.

**Methods:**

Between September and October 2020, ten focus group discussions with PrEP service providers from both hospitals and KPLHS across Thailand were conducted of which there were six hospitals, one health service center, three KPLHS. All interviews were recorded and transcribed *verbatim* to identify providers’ experiences, attitudes, and perceived barriers regarding PrEP service delivery in Thailand.

**Results:**

Among the 35 PrEP service providers, most of them reported positive attitudes toward PrEP and believed that it is an effective tool for HIV prevention. Men who have sex with men were perceived to be the easiest group to reach while PrEP uptake remains a challenge in other key populations. Integration of a PrEP clinic with other HIV services at hospitals made most healthcare providers unable to adopt an active approach in recruiting new clients like at KPLHS settings. Challenges in delivering PrEP services included lack of public awareness, high workload, limited benefit package coverage, structural and human resources.

**Conclusion:**

Additional services to address different health needs should be considered to increase PrEP uptake among harder-to-reach populations. Novel approaches to PrEP service integration and close collaboration between hospitals and KPLHS would be essential in optimizing PrEP uptake and retention. Support regarding raising awareness, expanding service coverage and access, improving facilities and workforce, and providers’ capacities are crucial for the success of the national PrEP programme.

## 1 Introduction

In the past three decades, Thailand has made remarkable progress toward Human Immunodeficiency Virus (HIV) treatment and prevention. These include the “100 Percent Condom Program” among commercial sex workers in 1991 (1) and the country’s clinical trials of antiretroviral therapy (ART) to prevent mother-to-child HIV transmission (2). This has culminated in a significant reduction in HIV incidence rates and made Thailand the first country in Asia to eliminate new HIV infections among newborns in 2016 (1, 3). In 2005, the Thai government included ART into the Universal Health Coverage (UHC)‘s benefit package, followed by compulsory licensing for the antiretroviral drugs (ARV), efavirenz (EFV) and the lopinavir/ritonavir (LPV/r) ARV combination in 2006 and 2007, respectively (4).

Despite numerous achievements, there has been slow progress in scaling up the service. HIV prevalence has been increasing among key populations who have high risk of HIV infections, including men who have sex with men (MSM), transgender women (TGW), sex workers (SWs), and people who inject drugs (PWID) while the proportion of people living with HIV (PLWHIV) who achieved virological suppression is still relatively small (5, 6). In 2021, with the total population of 71.6 million, it has been estimated that there were around 530,000 PLWHIV, 6,500 new HIV infections, and 9,300 HIV-related deaths in Thailand (7). HIV prevalence among adults aged 15–49 years old was around 1% (7) with higher proportions among individuals who are at high risk: 1.1% among female sex workers, 3.8% among male sex workers, 4.2 among TGW, 7.3 among MSM, and 7.8% among PWIDs (8). As part of the country’s commitment to end AIDS epidemic by 2030 following the World Health Organization (WHO) and the Joint United Nations Programme on HIV/AIDS (UNAIDS)’ global strategy initiative, the Thai government has set a number of priorities within the National AIDS Strategic and Operational Plan including expanding ARV access to all HIV-infected Thai nationals regardless of CD4+ level (9) and initiating the first fee-based HIV pre-exposure prophylaxis (PrEP) program in 2014 (10). Since then, PrEP has been an essential element of the national combination prevention package and included into the UHC in 2019 (5, 11). PrEP service in Thailand can be provided either from healthcare providers at hospitals or at Key Population Led Health Services (KPLHS) where the service is usually delivered by trained lay providers who are members of key populations. The KPLHS model was established in 2015, under the context of KP-leadership which means the services addressing key health issues identified KP members were provided under a “needs-based, demand-driven, and client-centred” approach to ensure non-judgmental and stigma-free environment (12–14). Currently, KPLHS provide the majority of PrEP services accounting for 82% of PrEP users in Thailand (15). The number has increased more than double since the introduction of KPLHS in 2019.

In 2020, the National Health Security Office (NHSO) launched a pilot project to provide PrEP for 2,000 new clients at 50 PrEP service centers across the country. To date, few qualitive studies have been conducted on PrEP services and are largely based on patients’ perspective (16, 17). This qualitative study was conducted to explore insights of service providers across Thailand from two PrEP service delivery models (hospital and KPLHS settings). As a part of the national monitoring and evaluation framework to evaluate early adoption of the country’s PrEP service under the UHC, this qualitative study aims to describe experiences and barriers concerning PrEP initiation and retention under service providers’ perspectives from both hospital and KPLHS settings as well as their foreseeable challenges in scaling up PrEP service and suggestions to overcome them. This is critical to inform the improvement of the national PrEP service and the development of a combined HIV prevention package, especially among individuals at high risk and key populations.

## 2 Methods

### 2.1 Study settings

We conducted 10 focus group discussions (FDGs) with 35 service providers from 10 PrEP centers participating in the NHSO’s PrEP pilot project: 6 hospitals, 1 health service center, 3 KPLHS. These facilities were from 5 provinces (Chiang Mai, Udonthani, Songkla, Ratchaburi, and Bangkok) across 4 regions in Thailand. The study was reviewed and approved by the Research Ethics Committee, Faculty of Public Health, Chiang Mai University (Document No. ET017/2020).

### 2.2 Data collection

We conducted focus group discussions from September to October 2020 using a semi-structured interview guide developed by the research team to capture further insights not covered in the quantitative self-administered online survey (18). The earlier quantitative study included service providers from all 50 active PrEP centers across the country (18). The main content of the predetermined interview guides is presented in Table 1.

**Table 1:** Interview guides.

When did your center start providing PrEP service? Why did your center decide to participate as a PrEP service center? What are the sources of funding for PrEP service at your center? How many personnel are responsible for PrEP service at your center? Who are they? How often does your center provide PrEP service? How many days per week? Do you think this is too much or too little? What is the service delivery model used by your center? What are the pros and cons to the use of this model? Who are your main clients? How do you generally recruit PrEP clients at your center? Which population groups are the most difficult to reach for your center? Why? How does your center perform in terms of recruitment and retention? How do you work with hospitals/KPLHS in your area? In your opinion what improvements are needed to fill gaps in PrEP service delivery? How do you evaluate the candidate’s eligibility if they want to take PrEP? Do you think the criteria should be made easier or harder? Why? How do you monitor and follow up on your PrEP clients? What are challenges and barriers in providing PrEP service? What kind of support would be helpful to improve the PrEP programme?

A purposive sampling was used to recruit participants from PrEP service centers with over 10 active PrEP clients and 1 year of experience from different service delivery models and geographical settings. Eligibility criteria included presently providing PrEP service at either hospital or KPLHS with PrEP clients’ engagement for over 6 months. All participants were contacted through their center contact lists, provided by the Center for Disease Control and Prevention, Ministry of Public Health (MoPH) and screened for eligibility. Sample size was determined by saturation of information, and extensive discussions with the study team members which would be finalized by the principal investigator (SC) who has extensive experience in this area.

Each focus group was comprised of 2 to 6 service providers working at the selected PrEP centers. All interviews were conducted in Thai either face-to-face or virtually led by the principal investigator. Interviewers explained about the study, objectives, and asked for participants’ consent verbally before starting the interview. Each interview lasted between 60 to 120 min and was recorded. The audio recordings were transcribed *verbatim*. Each participant was compensated with 300 Thai Baht ($8.66 USD) for their time.

### 2.3 Data analysis

Analyses of transcripts to identify providers’ perceived barriers concerning PrEP services and PrEP users’ perceptions were reviewed and summarized by two project team members in which disagreements were resolved through discussion and consensus. Braun and Clarke’s thematic approach involving six steps (familiarization with the data, generating codes, searching for themes, reviewing themes, defining and naming themes, and locating exemplars) was applied where the data was initially given a new thematic code without having to fit any pre-existing themes. The themes later emerged and were linked through the data (19). Transcription relating to barriers and key success/failures concerning PrEP implementation were listed. The completed list of categories and data extraction were reviewed by an additional investigator for clarification, presentation, and detail. Then the final list and transcripts were summarized for write-up.

## 3 Results

### 3.1 Respondent characteristics

Ten focus groups were conducted among 35 service providers engaging in PrEP services from Chiang Mai, Udonthani, Songkla, Ratchaburi, and Bangkok. Among these, 21 participants were from hospital or government health service centers whereas 14 were from KPLHS which were Rainbow Sky Association of Thailand (RSAT), Service Workers in Group (SWING), and MPlus (Table 2). More than half of the participants reported as female (54.3%) while 45.7% reported as males. Forty percent of the participants were PrEP counselors from KPLHS settings (14 of 35) whereas all participants from hospitals were reported as healthcare practitioners (HCPs) of which the majority were nurses (16 of 35; 45.7%), followed by physicians (3 of 35; 8.6%) and pharmacists (2 of 35; 5.7%) respectively.

**Table 2:** Respondent demographics.

Sociodemographic characteristics (*N* = 35)	Overall (%) (*N* = 35)
Settings	
Hospital	17 (48.57)
Health service center	4 (11.43)
Key Population Led Health Service (KPLHS)	14 (40.00)
Gender	
Male	16 (45.71)
Female	19 (54.29)
Primary occupation role	
Nurse	16 (45.71)
Physician	3 (8.57)
Pharmacist	2 (5.71)
Others, e.g., PrEP counselor/coordinator, KPLHS manager	14 (40.00)

^*^At KPLHS, PrEP counseling is generally provided by trained lay providers who are not HCPs whereas blood collection/sampling is done separately by technicians who are not engaged in PrEP counseling.

### 3.2 Qualitative findings

Five thematic areas emerged: Decision to engage in PrEP service, service delivery, clients’ recruitment, monitoring and retention, and challenges and recommendations to improve PrEP program. The key insights of each thematic area are detailed below.

#### 3.2.1 Decision to engage in PrEP service

The main reasons to participate as a PrEP service center were high-HIV prevalence, high proportion of key population who are at high risk, no other PrEP center in the area, and proximity to key populations. Some hospitals were selected to join the pilot program because they had experience in offering HIV and sexual transmitted disease (STD) services with availability of ARV clinic in place. Most providers reported that PrEP service was an effective tool to prevent HIV transmission:

*‘PrEP is another tool apart from condoms to prevent HIV infection’*. (Male PrEP counsellor).

*‘We have many HIV cases. Prescribing medication for prevention should be better than taking ARV’*. (Female HCP).

*‘The center was selected to join the pilot program because we are close to BTS [convenient for key populations]’*. (Female HCP).

#### 3.2.2 Service delivery

At the hospital setting, PrEP service was generally integrated with HIV Voluntary Counseling and Testing and ARV clinic while at KPLHS, PrEP clinic was usually run separately. All KPLHS offered same day PrEP service where providers dispensed PrEP medication to the clients on the first visit date and PrEP users would be later contacted in case there were any abnormal laboratory findings while some hospitals dispensed PrEP on the next day after obtaining the laboratory results. This was raised as a limitation in recruiting new clients by some providers:

*‘The distinction between two service delivery models is that at hospital, we have limited medication supplies unlike at KPLHS that PrEP can be offered on the same day without having to wait for lab results [which is more convenient]. On the next day, patients may not come back’*. (Female HCP).

Many providers noted ‘same day PrEP’ as an advantage in reaching and recruiting new cases:

*‘Same day PrEP helps to close the gap and make PrEP more accessible to potential clients’*. (Male PrEP counsellor).

Most KPLHS started their PrEP clinic in the afternoon until evening while majority of hospitals’ PrEP clinic operated during office hours. The clinic’s working hours were also crucial to make the service accessible to clients, especially key populations:

*‘It takes a long time to wait at the public hospital. The operation time [which is the office hours] is not convenient for clients [who are key population]’*. (Male HCP).

*‘Apart from convenience, visiting the clinic after office hours can also avoid stigmatization’*. (Male HCP).

Both hospital and KPLHS providers agreed that pharmacies might not be appropriate for PrEP provision due to their concerns regarding venue space to allow privacy and confidentiality and monitoring of laboratory tests:

*‘VCT [Voluntary Counseling and Testing] is needed for screening. I am concerned about how this will be done at pharmacies’*. (Male KPLHS provider).

*‘It is possible [to have PrEP delivered at pharmacies] among those who have good adherence. However, I think they’d prefer [receiving PrEP] at hospital because of their concern regarding confidentiality’*. (Female HCP).

*‘[At pharmacy], it can be double-edged sword as we do not know their blood testing results. It may be fine for refilling medication, but clients still need to visit hospital to follow up on their laboratory tests…’*. (Male HCP).

#### 3.2.3 Clients’ recruitment and target PrEP users

**Figure 1:**
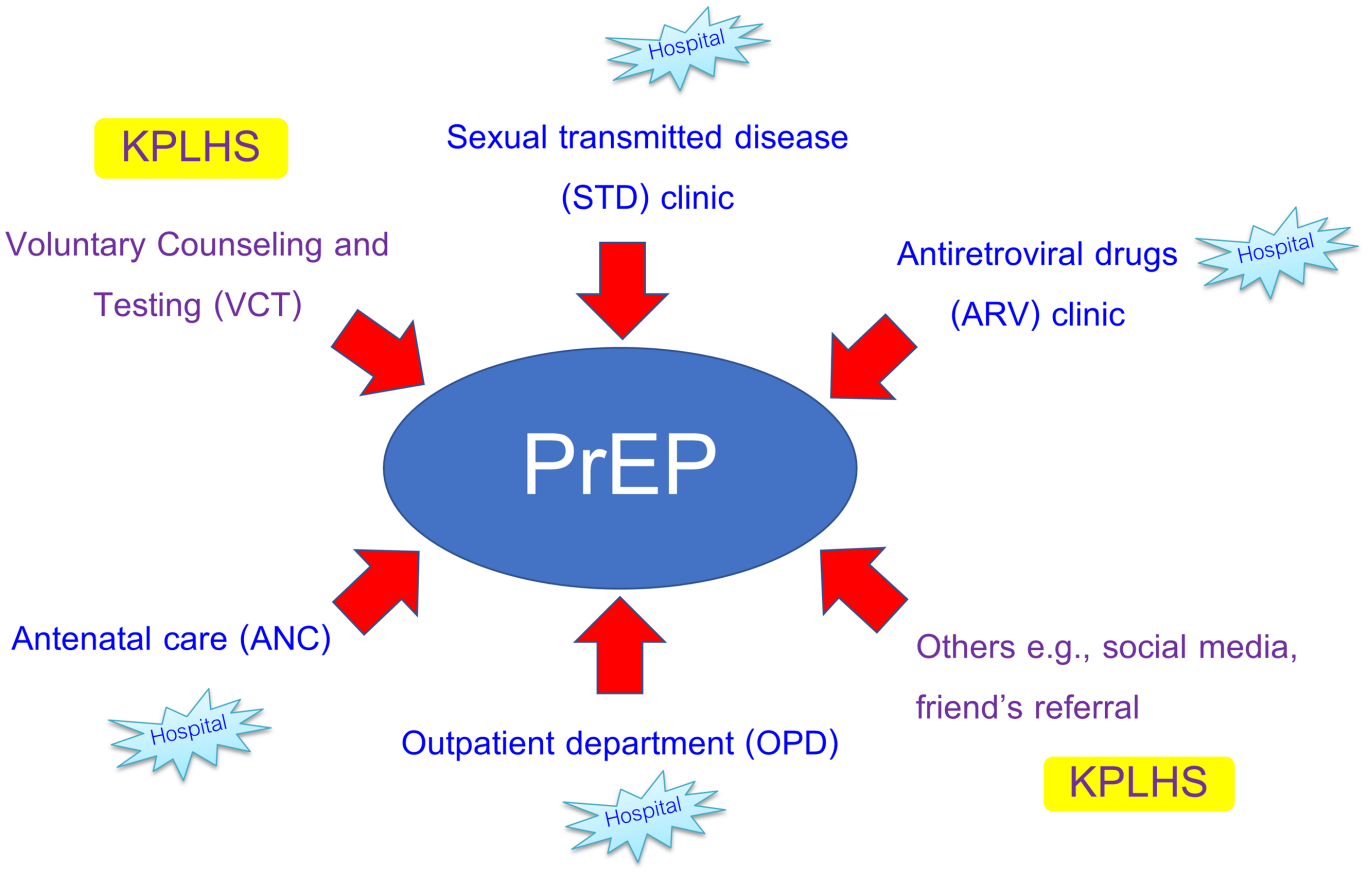
Channels for PrEP client recruitment between hospital and KPLHS settings.

Different PrEP client recruitment strategies were employed (Figure 1). PrEP clients were referred from hospital STD/ARV, and antenatal care (ANC) clinics as well as recruited from KPLHS via Voluntary Counseling and Testing (VCT), friend’s referral and social media platforms. PrEP counselors at KPLHS actively screened and identified high-risk populations through mobile VCT and multiple social media platforms while healthcare providers at hospitals generally adopted a passive approach preferring potential PrEP users to present themselves at the facilities or having them referred from other clinics, e.g., STD/ARV clinic and ANC. Few hospital providers mentioned their involvement in mobile VCT with limited success as many potential PrEP users were concerned about HIV testing and were unprepared to know their infection status:

*‘We’ve been to many places [to promote PrEP] including military barracks, 5 schools/vocational institutions, and juvenile observation and protection centers. The only places left [that we have not been to] are entertainment venues [but no one was interested in taking PrEP]’*. (Female HCP).

*‘Some people are afraid to have their blood taken for HIV testing. When they were told that they needed to undergo HIV testing [before being prescribed PrEP], they said no.’* (Female HCP).

Sex workers and people who use drugs were perceived to be the most difficult to reach groups. Many providers expressed challenges in reaching these populations:

*‘The form of commercial [sex] service has been changed. They are now online instead of physically available at entertainment venues [so it is harder to reach sex workers for Voluntary Counseling and Testing].’* (Male PrEP counsellor).

*‘We tried to convince all cases we have at drug addiction clinic to take PrEP but no one was interested’*. (Male HCP).

*‘People who use drugs are the most unprepared [among all high-risk groups]. They are not ready to cooperate and adhere to medication. It is also not convenient for them to come to hospital’*. (Female HCP).

*‘Currently, we do not have any PrEP users who are drug users. They are a very closed group [not open to any outsiders]’*. (Female HCP).

Apart from that, some providers reported having new clients through friends’ referrals and individuals who were HIV Post-exposure Prophylaxis (PEP) users:

*‘We will tell them [PEP users] that it is the continuing program from PEP to PrEP among those who are interested. We will convince most of our PEP clients to continue taking PrEP’*. (Male PrEP counsellor).

*‘I normally inform my clients that they will be prescribed PrEP after taking PEP medication and ask whether they agree with that. This is because they are at high risk and may continue having that risk [of HIV infection]. That’s why they take PEP. If they are ok, I will dispense PEP. It is like we have an agreement together’*. (Male PrEP counsellor).

Most PrEP users at hospitals were heterosexual individuals who had HIV-positive partners or were referred from other clinics including STD and ANC. On the contrary, most clients at KPLHS were from key populations mainly men who have sex with mem (MSM) individuals followed by transgender women (TGW). Most KPLHS providers felt that MSM were the easiest to reach group as they generally had good knowledge about PrEP and perceived themselves as having a high risk of HIV infection.

*‘MSM are the easiest. They are the majority of our clients. They are knowledgeable, educated and have a wide network where they also refer their friends to take PrEP. Taking PrEP is even a plus to their profiles in some online dating applications’*. (Male PrEP counsellor).

Most counselors recognized TGW as a more difficult to reach group due to their usage of hormone therapy which might be a barrier to concomitantly take PrEP in the long term:

*‘TGW are afraid that they may forget to take PrEP because they take a lot of medications [including hormone therapy] concomitantly and have concern that they cannot adhere to medication’*. (Male PrEP counsellor).

Some KPLHS counselors mentioned that TGW perceived that they had lower risk for HIV acquisition compared to MSM:

*‘TGW have undergone sex reassignment surgery. They have sexual intercourse through their vagina and consider themselves to have lower risk [compared to MSM] in acquiring HIV infection’*. (Male PrEP counsellor).

In contrast, healthcare providers from hospital settings who also provided PrEP to inmates discerned TGW in prison to be an easier to reach population as they did not take any hormone therapy and felt comfortable talking to female healthcare providers:

*‘TGW are easier to reach [compared to other key populations]. They see us as their sisters’*. (Female HCP).

#### 3.2.4 PrEP clients’ monitoring and retention

Most participants reported that over half of their clients could stay on PrEP for over 6 months of which the majority were MSM, followed by negative partners of serodiscordant couples. At hospitals, PrEP users were usually followed up according to routine practice without any reminders/frequent contacts whereas at KPLHS, PrEP clients were followed up more closely through online platforms:

*‘Patients can book their appointments through the website [take me now] and they will get SMS to confirm their appointments’*. (Male KPLHS provider).

*‘We do not have any reminders, but we phone them if they do not show up at their follow-up visit’*. (Female HCP).

*‘We also have line official where we work with the responsible pharmacist to respond to their inquiries regarding medication(s)’*. (Male HCP).

The main reasons for loss to follow-up reported were relocation and changes of sexual risk behaviors:

*‘Relocation of their workplace is one of the major problems that make them (PrEP users) cannot come for follow-up visit’*. (Female HCP).

Most providers expressed the sentiment that maintaining good relationships and rapport with clients was essential for retention.

*‘Building good relationships is key to engage them to come back for follow-up’*. (Female HCP).

#### 3.2.5 Challenges and recommendations to improve PrEP program

Many providers from hospital settings expressed the lack of structural resources and manpower as significant issues hindering PrEP service delivery. Integration of PrEP service with ARV, STI clinics and Voluntary Counseling and Testing at hospital settings had culminated in high workloads which impeded active screening and counseling of high-risk individuals for PrEP initiation and retention:

*‘At hospital, we have limited manpower, so the provider-client relationship is not as close as at KPLHS’*. (Female HCP).

*‘Our center is a two-storey building which has very limited space but serving over a thousand of patients’*. (Male HCP).

*‘The service at hospital usually has very limited space which does not allow enough privacy [for key population]’*. (Male KPLHS provider).

Most providers felt that PrEP was not widely known and there was a need to raise awareness about PrEP among healthcare providers and the general population:

*‘PrEP is something new to healthcare providers who are not involved in PrEP. Even hospital staff who are not involved [in PrEP] do not know anything about it. We need to raise awareness and educate HCPs more’*. (Female HCP).

*‘Very few people know about PrEP and have access of which most users are MSM. Promoting and publicizing [PrEP] might have been done too little. PrEP is not available at all hospitals. Therefore, access is limited in some settings. As many people are not aware about PrEP, when we promote it to outsiders, we can only get some certain groups and cannot make it accessible to a wider population’*. (Female, HCP).

Notably, some providers were concerned about the perception of other healthcare providers regarding PrEP that it might result in sexual risk compensation:

*‘[Some] doctors disagree to use PrEP. They think this will make clients less likely use condoms’*. (Female HCP).

Healthcare providers additionally expressed their high workload concerning data entry in different programs and called for a single system to avoid repetitions of work and overlapping data as well as the need for condoms and lubricant supplies:

*‘The programs were complicated, and I had to work outside of office hours to enter similar information’*. (Male HCP).

*‘Condoms are sufficient for the size that we do not want (49″) while the size that we want (52″, 54″, and 56″) are not provided’*. (Female HCP).

To increase PrEP uptake, healthcare providers suggested a seamless process to allow same day PrEP and a referral system in case of clients’ relocation:

*‘[I] would like the hospital to have more supplies in our stock so that we can dispense PrEP right away […] Then we only phone them in case of abnormal laboratory findings such as […]. This should be more convenient for clients as they may not come back to be informed about the results of their blood test’*. (Female HCP).

To increase PrEP uptake among TGW, some providers suggested having additional services such as feminizing hormone therapy counseling for TGW and emphasized the need for training of PrEP providers to support TGW’s specific health concerns:

*‘There should be educational materials concerning feminizing hormone therapy and its potential drug interactions [with PrEP] for TGW’*. (Male KPLHS provider).

Finally, when asked about prioritizing groups to receive PrEP, participants reported that PrEP should be given to all high-risk individuals or any person who would like to take it including a non-Thai citizen:

*‘PrEP should be available to all who want to take it even non-Thai citizens as [some] of these individuals also have Thai partners. In case we do not do well for preventive measures, this can also be the burden of our health system’*. (Female HCP).

## 4 Discussion

The aim of this study is to understand how to improve the national PrEP service and overcome foreseeable challenges in scaling up PrEP services in Thailand. Our findings have identified themes which have promising implications for improvement and expansion of the PrEP program among key populations and individuals at high-risk. Overall, most service providers reported positive attitudes toward PrEP and perceived it as an effective tool for HIV combination prevention program. This is consistent with the previous quantitative study (18). However, challenges remain at the micro-level management and service integration with opportunities to scale up the service among key populations.

Differences between the delivery model used at KPLHS and the model used at hospital settings have significant impact on their PrEP service operation and approach in reaching new clients. KPLHS’s primary focus on PrEP allows KPLHS providers to adopt an active approach in reaching and recruiting potential new users through VCT and mobile PrEP. In addition, extensive PrEP promotion through social media/online platforms and availability of ‘same day PrEP’ at KPLHS are also their key success factors in recruiting many new clients. On the contrary, the integration of PrEP service with HIV and STD services at hospital settings may impose a high workload on healthcare providers making them prefer a more passive approach which may be ineffective for PrEP uptake. More collaboration between hospitals and nearby KPLHS for cases’ referral and recruitment is essential for a seamless process and expansion of PrEP service access.

The hours that PrEP service is available is also important in engaging potential clients. Flexible operation times at KPLHS makes the service more accessible among key populations compared to hospitals where PrEP service is usually provided only during office hours. According to some service providers in our study, accessing PrEP service outside office hours is a feasible way to avoid stigmatization. HIV-related stigma and discrimination have been a significant obstacle to health services access leading to poor disease outcomes (20). HIV-stigma is also negatively associated with mental health and health-related quality of life (HRQoL) (20). Negative self-image or internalized stigma has been noted as the cause to avoid visiting a healthcare facility by one-third of people living with HIV (21). In our study, fear of knowing HIV serostatus has been reported as a key obstacle in reaching key populations for VCT which could impede PrEP uptake. This emphasizes the need for social empowerment and interventions to eliminate HIV-related stigma particularly negative self-image which has been recognized as more impactful than external stigma (22).

**Figure 2:**
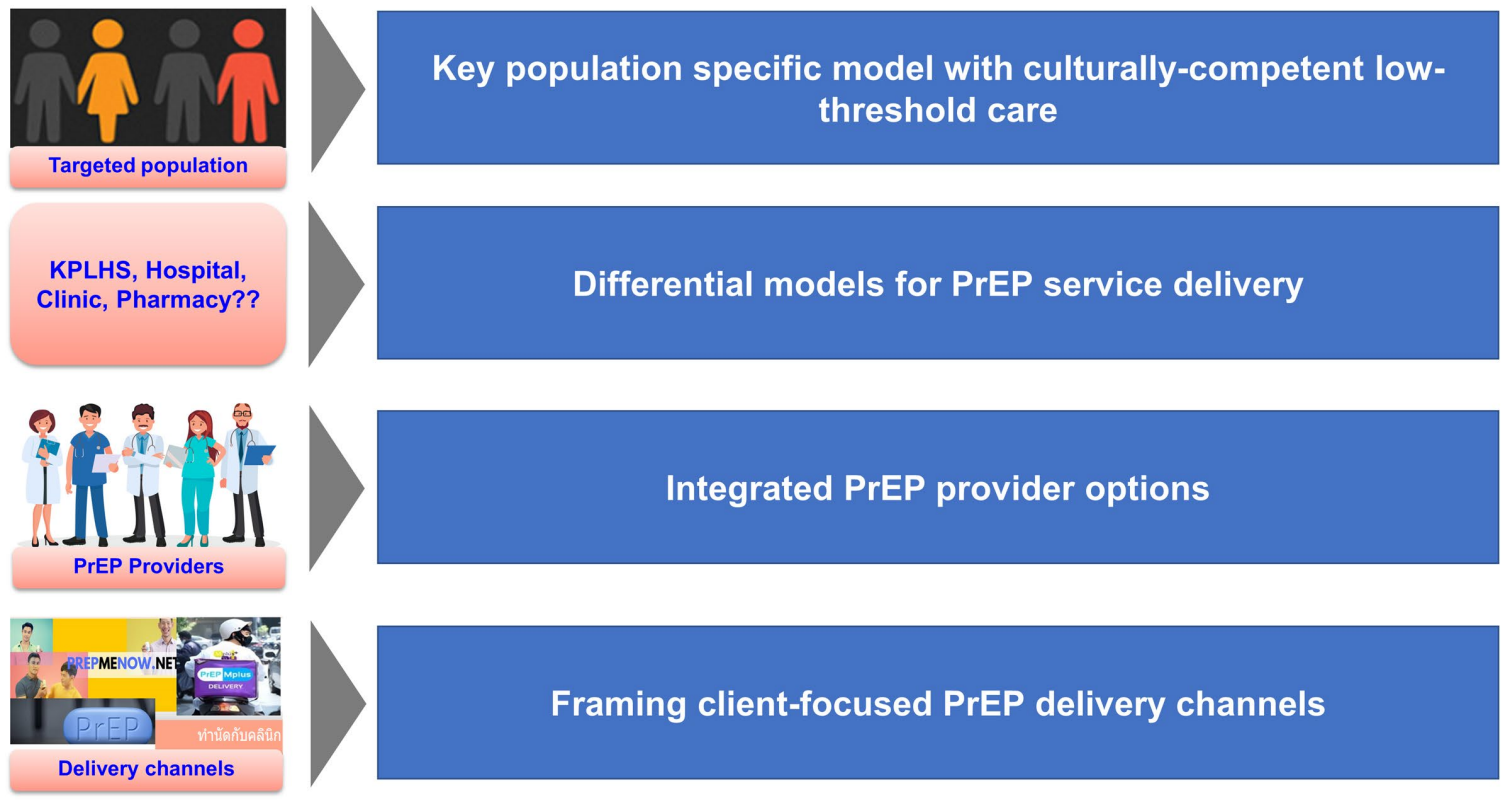
Novel approaches for differentiated PrEP service delivery.

Differential PrEP service delivery models with client-focus are essential to enhance HIV-prevention efforts (Figure 2). Despite the availability of PrEP service to all populations at risk in Thailand, access to service is still a problem for some key populations especially transgender women and people who use drugs who may have specific health needs. Transgender women’s desire for additional services based on their needs were noted in a number of previous studies (23, 24). Although clinical data in Thai transgender women suggested no significant drug–drug interactions between female hormone therapy and PrEP that would impact on PrEP protection level (25), this is still a major concern that has hampered the uptake and adherence of PrEP among this population. Additional services to address female hormone therapy as well as sensitizing and gender affirming therapy trainings to strengthen capacities of healthcare providers and KPLHS providers to better serve this group of clients are needed to increase PrEP uptake among transgender women. In response to the unmet health needs of TGW population, the first transgender-led sexual health clinic in Thailand named “Tangerine Community Health Clinic” was established in 2015 to offer comprehensive healthcare services through lay providers who are members of transgender community and gender sensitive healthcare providers. Up to March 2021, Tangerine Clinic has served over 3,900 TGW (26) with significant reduction in the annual HIV prevalence and syphilis from 2016–2019 (27). This stresses the imperative role of this specialized service model as a key component of HIV prevention to achieve HIV epidemic control.

All service providers in our study reported they had no PrEP experience in dealing with people who use drugs. Our respondents consistently remarked that people who use drugs were the most difficult to reach population due to being unprepared for medication adherence and the inconvenience of visiting PrEP clinics. Integrating PrEP services into existing access venues such as methadone maintenance programs which already require routine behavioral counseling in place may have a profound impact on HIV prevention efforts. Long-acting PrEP formulations such as injectable carbotegravir (CAB-LA) which can be given once every 8 weeks can be a viable option to improve uptake and retention among this population whose adherence to daily oral PrEP imposes significant challenges.

Community pharmacies have been suggested as promising additions to the delivery of PrEP services to reduce the burden on the rest of the PrEP service system. This has been well accepted in the USA (23, 28). However, both hospital and KPLHS providers reported that PrEP delivery at pharmacies might not be exclusive enough to enable privacy for PrEP provision. Some providers also raised their concerns about the issue regarding blood collection for routine laboratory monitoring which may not be viable at pharmacies. As novel approaches are needed to expand service access, providing PrEP at pharmacies may be a potential alternative under the service integration model where PrEP is delivered by trained pharmacists working in partnership with hospitals.

Several major challenges were raised by healthcare providers from hospital settings. The repetition of work requiring data entry into different programs is one of the main causes of their excessive workload outside office hours. Therefore, a single user-friendly system for PrEP program data entry regardless of sources of fundings should be considered to enable efficient operation and monitoring and evaluation processes. Limited space for service delivery could compromise privacy for PrEP provision, emphasizing the need to improve infrastructure and healthcare facilities. The limited coverage of the UHC’s benefit package for laboratory tests, insufficient condoms, lubricant supplies, and medications remain challenges in PrEP service delivery. Increasing coverage for some specific cases that require additional laboratory tests, sufficient medications and supplies are essential to enable same-day PrEP and enhance service efficiency. The limited-service coverage for non-Thai populations is also a barrier that hampers the country’s ambitious goal to end HIV/AIDS by 2030. Thus, expanding health services access as well as education to improve the public’s attitude about migrants as a “temporary source of labor” (29) are needed.

Although respondents reported good knowledge about PrEP and none of them reported increases in risky behavior among their clients, most of them agreed that there was a need to raise awareness among HCPs who are not involved in PrEP. This awareness should include an improved understanding that PrEP has not led to an increase in high-risk behavior. HCPs at hospital settings are in a position to counsel and refer potential clients for PrEP initiation. A lack of HCPs’ understanding has been associated with never having HIV testing (30), increased sexual behavior risk (31), and reduced healthcare service utilization (32). Therefore, educating HCPs outside PrEP service is crucial to improve efficiencies of service integration and facilitate patients’ referrals to PrEP uptake.

This study provides comprehensive insights from PrEP service providers in Thailand from both hospital and KPLHS settings. However, there are several limitations in our study to be noted. As this is a qualitative study, the data produced might be subjective and may impose transferability limits. Most of the study participants were experienced with good knowledge about PrEP. Therefore, the generalizability may be limited and may not be applicable for settings at the early stage of PrEP implementation. We did not collect the information concerning sexual orientation and age of the participants as the main purpose of the study is to collect insights and challenges regarding PrEP service in Thailand. The use of thematic analysis in this study can impose challenges concerning differentiations between codes and themes where different types of themes could be generated. However, this also allows more flexibility and application of researchers’ experiences to gain deeper understanding of the insights. Finally, to get different points of view, we included multiple staff involved in PrEP from the same center in each focus group. Hence, it is possible that the influence of different hierarchies and powers might have undermined candid conversations. Nevertheless, we did not identify any significantly different opinions or attitudes among participants within the same setting in the analyses.

## 5 Conclusion

Most service providers have positive attitudes and good knowledge about PrEP service in Thailand. Despite success in HIV combination prevention and PrEP service implementation, large opportunities to scale up remain among key populations. The innovative PrEP service delivery model that focuses on clients’ specific needs is essential to increase PrEP uptake among harder-to-reach populations. The KPLHS reach-recruit to hospital model should be adopted to optimize PrEP uptake. Support regarding raising awareness, improving facilities and human resources, service coverage and providers’ capacities are important for the success of the national PrEP programme.

## Data availability statement

The original contributions presented in the study are included in the article/supplementary material, further inquiries can be directed to the corresponding author.

## Ethics statement

The studies involving humans were approved by the Research Ethics Committee, Faculty of Public Health, Chiang Mai University. The studies were conducted in accordance with the local legislation and institutional requirements. The ethics committee/institutional review board waived the requirement of written informed consent for participation from the participants or the participants' legal guardians/next of kin because this is a focus group discussion among service providers. There is no involvement of any patient information or personal information. The study participants provided verbal informed consent before completing study procedures. Written informed consent for participation was not required for this study in accordance with the national legislation and the institutional requirements.

## Author contributions

AR: Conceptualization, Data curation, Formal analysis, Investigation, Methodology, Project administration, Resources, Software, Validation, Visualization, Writing – original draft, Writing – review & editing. SiC: Data curation, Project administration, Writing – review & editing. KI: Data curation, Writing – review & editing, Project administration. CC: Project administration, Resources, Writing – review & editing. PK: Funding acquisition, Resources, Writing – review & editing. DO: Supervision, Writing – review & editing. SuC: Conceptualization, Funding acquisition, Resources, Supervision, Writing – review & editing.
